# Ten-year outcome of a dedicated hip fracture unit embedded within a level 1 major trauma centre

**DOI:** 10.1308/rcsann.2024.0094

**Published:** 2025-04-02

**Authors:** B Ahmad, F Davis, G Chan, BA Rogers

**Affiliations:** ^1^ Brighton & Sussex Medical School, UK; ^2^ University Hospitals Sussex NHS Foundation Trust, UK

**Keywords:** Hip fractures/femoral neck fractures, Treatment outcomes, Mortality

## Abstract

**Introduction:**

Fragility hip fracture (FHF) care has been revolutionised by the introduction of the best practice tariff and its associated quality assessment domains. However, increasing demands on trauma services, most notably in regional major trauma centres (MTC), have resulted in competing challenges for clinical resources. This study aims to identify whether a dedicated hip fracture unit (HFU) embedded within a trust hosting the regional MTC affords sustained improvements in clinical outcomes for FHFs.

**Methods:**

A 10-year retrospective cohort study was performed using a prospectively collected database that was cross-referenced against contemporary data submitted to the National Hip Fracture Database by a single multicentre National Health Service trust. The study period encompassed a 10-year period covering 5 years before and 5 years after formation of a dedicated HFU. The outcomes evaluated include time to surgery, length of hospital stay, discharge location and mortality.

**Results:**

Some 4,998 patients sustained FHFs: 2,387 patients (2,533 injuries) treated prior to formation of the HFU formation and 2,611 patients (2,813 injuries) treated after. The mean time to surgical intervention was significantly lower in the group treated at the HFU by 3.1 days (*p *< 0.001). Length of hospital stay was also significantly reduced at the HFU (*p *< 0.001). More patients were discharged back to their premorbid residence from the HFU (47% vs 40%). The 30- and 365-day mortality rates were significantly reduced at the HFU (*p *= 0.005 and *p *= 0.024, respectively).

**Conclusion:**

When embedded within an MTC, the HFU model is a sustainable operational structure in the medium term that provides clear clinical benefits and could be replicated nationally and internationally at similar institutions.

## Introduction

Fragility hip fractures (FHFs) affect ∼72,000 patients per annum in the United Kingdom (UK) with this number expected to rise exponentially with the ageing population.^1,2^ The increasing incidence will place greater demands on both acute hospital and community care services.

Walton *et al* demonstrated that a dedicated ‘hip fracture unit’ (HFU) embedded within a level 1 major trauma centre (MTC) resulted, in the short term, in reduced variance in overall length of stay (LoS) and trends suggesting a reduced time-to-theatre and 30-day mortality.^3^ The HFU analysed in this study provided a centralised unit with ring-fenced theatres, and rehabilitation beds with dedicated co-located multidisciplinary teams (MDTs) incorporating physiotherapists, occupational therapists, surgeons and physicians. This HFU operated independently from the general trauma caseload of the MTC.

In 2012, trauma services in England were reorganised into a ‘hub-and-spoke’ model formed around 22 designated MTCs, centralising care for physical injuries that can result in death or severe disability.^4^ Morgan *et al* suggest a significant subsequent improvement in clinical care and outcomes for major trauma patients as a result the centralisation of expertise for the most critically injured individuals.^5^ The centralisation of major trauma cases has increased care demands at MTCs with the potential to displace ‘non-major’ trauma cases from operating theatres.^6^ In 2022, four of the ten hospitals showing the lowest compliance with the best practice tariff’s (BPT) target of “surgery on the day of, or after, admission” were either MTCs or part of a wider organisation providing MTC services.^7^

Centralisation of FHF care has the potential to ensure timely multidisciplinary care with dedicated orthogeriatric and allied health professional input, both of which have been demonstrated to reduce length of hospital stay and mortality.^8^

This study aims to establish whether an HFU, compared with a non-dedicated unit treating a mixed major trauma and FHF caseload, has a sustained clinical and organisational benefit to support its implementation with other similar healthcare organisations.

## Methods

### Study design and population

A retrospective cohort study of all consecutive patients over a 10-year period presenting with a FHF eligible for BPT payment between 1 July 2010 and 30 June 2020 was performed using prospectively collected databases. National Hip Fracture Database (NHFD) records for University Hospitals Sussex NHS Foundation Trust (hereafter the Trust; formerly Brighton & Sussex University Hospitals NHS Trust) were examined and cross-referenced against local patient records.

Patients who sustained a FHF as part of a polytrauma presentation (Injury Severity Score ≥15) and those ineligible for BPT payment for any other reason were excluded from this study.

Patients were divided into two cohorts: pre-HFU and post-HFU formation. The HFU became operational on 1 July 2015.

### Study centres

The Trust was responsible for two hospitals. This first was the Royal Sussex County Hospital, which serves as south-east England’s level 1 adult MTC. All orthopaedic trauma theatres were located at the level 1 MTC prior to formation of the HFU. The second hospital was the Princess Royal Hospital, which functions as a district general hospital (DGH) serving its own discrete catchment area. Approximately 500–700 patients with FHFs were treated per annum, ranking the trust within the second decile of the NHFD for case volume.^7^

### Study outcomes

The primary outcome of this study was a comparison of the time to surgery between pre-HFU and post-HFU patients; time to surgery is a criterion assessed by both the BPT and the UK’s Care Quality Commission as a surrogate marker of a hospital’s overall performance.^9^ In addition, this study also assessed patients’ length of stay, 30-day and 365-day mortality in addition to length of stay (LoS).

### Study methodology

Local copies of NHFD records for the study period were reviewed to segregate patients into the pre- and post-HFU cohorts. Missing data points within the NHFD data set were referenced against local electronic patient records.

Dates of death for all patients included in the study were referenced against primary care records to provide a near complete data ascertainment.

Baseline characteristics for pre-HFU and post-HFU patients were compared to assess for differences in underlying demographics: age, gender, type of fracture and American Society of Anesthesiologists (ASA) grade. These are known risk factors for mortality, and potential causes of medical delays to patients attending theatre.

Patients who sustained fractures of both hips but at different time points, were included as two separate events in this study, and each fracture was analysed independently of the other.

### Intervention

The pre-HFU cohort comprised patients presenting (either via the emergency department or diagnosed after an inpatient fall) between 1 July 2010 and 30 June 2015. During this period, FHF patients were treated together with major trauma patients at the MTC, and after having their surgery they were transferred to the DGH to continue their convalescence prior to discharge. MDT input was provided at the MTC and DGH site, but there was no dedicated team to manage FHF patients.

**Figure 1:**
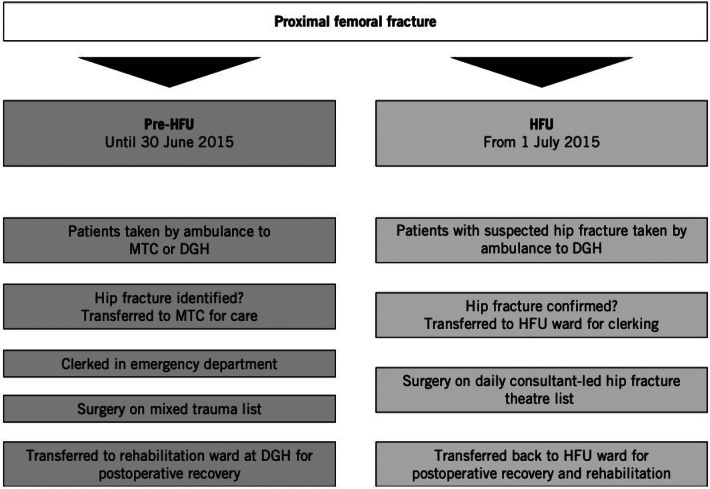
Schematic representation of the change in service provision following implementation of the hip fracture unit (DGH = district general hospital; HFU = hip fracture unit; MTC = major trauma centre).^3^

The HFU commenced on 1 July 2015 (post-HFU cohort) and was situated within the DGH and separate from the MTC. Patients were transported directly to the HFU if they were triaged as having a suspected hip fracture by the local ambulance service. Those patients who self-presented or were conveyed to the MTC and subsequently diagnosed with a FHF were transferred to the DGH for treatment. For the purposes of BPT and NHFD reporting, patients’ ‘treatment clocks’ started on their presentation to either of the two hospitals. Figure 1 outlines and compares the pathways between the two groups for a patient being admitted to hospital with a proximal femoral fracture.

The set-up of the HFU is detailed in our previous study (Figure 1), with the key features being^3^:

• prehospital protocol with ambulance service for suspected FHF
• coordinated transfer of confirmed FHFs to HFU
• reconfiguration of scheduled working hours for staff at HFU
• specialist rehabilitation ward
• dedicated daily consultant-led theatre lists.

### Ethical approval

Approval from the research ethics committee was not required because the data used for this study are routinely and periodically submitted to the NHFD as part of standard clinical practice and this was thus deemed a service evaluation study.

### Statistical analysis

Statistical analysis was conducted using Statistical Package for Social Sciences (IBM SPSS version 27.0). Fisher’s exact test was used to analyse categorical variables. For continuous variables Mann–Whitney U test was performed because the data were assumed to be non-parametric. The assumption for a non-parametric distribution was made on the basis that patients are more likely to experience delays in their time-to-theatre, with hip fracture mortality demonstrating a positive skew.^7^ The equality of variances was assessed by performing Levene’s test, which tested for a difference in the variance of the baseline characteristics between the pre-HFU and post-HFU cohorts to assess for the risk of bias from confounding factors.

Statistical significance for the analysis was set at *p *< 0.05.

## Results

### Study cohorts

During the study period, there were 5,346 FHFs sustained by 4,998 patients, all of which were included in this study. In the pre-HFU group there were 2,387 patients in whom 2,533 fractures were reported; for the post-HFU group there were 2,611 patients who sustained 2,813 fractures. There were no significant demographic differences between the two cohorts in terms of age, gender, fracture type and ASA grade (*p *> 0.05) (Table 1).

**Table 1:** Demographics of patients and intraoperative information

Study population	Pre-HFU	Post-HFU
Total patients, *n* (%)	2,387	2,611
Total injuries, *n* (%)	2,533	2,813
Mean age, years (SD)	82.8 (±9.7)	83.0 (± 9.4)
Sex (male : female)	698 : 1,835	834 : 1,979
Fracture type, *n* (%)		
Intracapsular – displaced	1,302 (51.4)	1,414 (50.3)
Intracapsular – minimally displaced	141 (5.6)	215 (7.6)
Extracapsular – intertrochanteric	993 (39.2)	1,069 (38.0)
Extracapsular – subtrochantericfracture	97 (3.8)	115 (4.1)
ASA grade, *n* (%)		
1	101 (4.0)	59 (2.1)
2	677 (27.0)	795 (28.6)
3	1,365 (54.4)	1,621 (58.2)
4	361 (14.4)	298 (10.70)
5	4 (0.2)	12 (0.43)

ASA = American Society of Anesthesiologists; HFU = hip fracture unit; SD = standard deviation from the mean

### Primary outcomes

The time from hospital presentation (or diagnosis after inpatient falls) to the start of surgical intervention was significantly lower in the post-HFU cohort than in the pre-HFU cohort (*p *< 0.001). The mean time to surgery for the pre-HFU group was 27.5h compared with 24.2h for the post-HFU cohort (Table 2).

**Table 2:** Results for pre-HFU and HFU groups

Outcome	Pre-HFU	Post-HFU	*p*-value
Time to surgery (h)			
Mean (SD)	27.5 (±20.76)	24.2 (±19.99)	<0.001
Length of stay (days)			
Mean (SD)	19.3 (±15.40)	16.5 (±12.20)	<0.001
Mortality (%)			
30-day	6.4	4.7	0.005
365-day	20.4	17.9	0.024

HFU = hip fracture unit; SD = standard deviation from the mean

### Secondary outcomes

The 30- and 365-day mortality rates following injury were significantly lower following introduction of the HFU than in the pre-HFU cohort. The 30-day mortality showed a 26.6% relative risk reduction, from 6.4% in the pre-HFU cohort to 4.7% in the post-HFU cohort (*p *= 0.005). The 365-day mortality rate demonstrated a smaller relative risk reduction of 11.8% from 20.4% to 17.9%, but this was still a significant change (*p *= 0.024) (Table 2).

Mean LoS was reduced, by an average of 2.8 days, after introduction of the HFU (pre-HFU mean 19.3 ± 15.40 days vs HFU mean 16.5 ± 12.20 days) (Table 2).

### Data ascertainment

Mortality data were not available for four patients who were all foreign nationals and were documented as having left the UK after their acute treatment for continued rehabilitation in their home countries. Data ascertainment was therefore >99.9%.

## Discussion

### Primary outcome

This study demonstrates that the short-term improvements in reducing variations of patients’ time-to-theatre (from hospital presentation) and the length of stay in hospital after sustaining a FHF with a dedicated HFU, are sustained in the medium term.^3^

### Secondary outcome

Our 10-year study of the impact of a dedicated HFU within a level 1 MTC demonstrates a significant reduction in 30-day mortality, to a rate below the national average. This improvement in short-term mortality rates is continued with 365-day survival, which is also substantially below the national average.

Formation of the HFU also demonstrated significant reductions in the LoS by nearly 3 days compared with the corresponding period prior to formation of the HFU, in concordance with the findings previously described by Walton *et al.*^3^

### Clinical efficiency

Time-to-theatre and LoS improvements not only provide benefits in patient outcome, but also benefits to the wider health economy. Reductions in length of stay demonstrate an expeditious journey through the treatment pathway, thereby increasing system capacity without the need to increase the overall number of inpatient beds and/or treatment capacity. This is pertinent in the current healthcare climate in which we are being asked to ‘do more with less’ with specific focus on streamlining patient pathways.^10^ Reductions in LoS have been shown to reduce the incidence of healthcare-acquired infections, pressure damage, cognitive decline and psychological disturbances.^11^

During the 5-year period reported after the HFU was set up, overall average LoS reduced by 2.8 days across a cohort of 2,813 episodes. This has the potential to equate to 1,575 bed days saved per annum, which would be equivalent to a reduction of the department’s bed base by four beds. Extrapolation across the national bed base occupied by FHFs is difficult because of local confounding factors, but the savings demonstrated in this single unit show the potential for exponential savings across the National Health Service (NHS) if a HFU model is adopted by similar organisations to that of the study centres.

The introduction of a ‘hub and spoke’ major trauma network has increased caseloads in those hospitals designated as MTCs.^12^ This has the potential to impact on the timely delivery of surgical care where operating theatre capacity has not increased to meet this uptrend in clinical demand. NHS England reports demonstrate a 5% increase in total operating theatre capacity (not including day case theatre capacity) during the post-HFU period, with most MTC trusts not showing any increase in capacity.^13^ This study has shown that the formation of dedicated HFUs with ring-fenced theatre capacity in pressurised MTC settings has the potential to ensure timely care of fragility fractures with no need to increase overall theatre capacity.

A time-to-theatre of <36h from presentation is assessed by the BPT and is the criterion with the lowest compliance rate.^14^ Although the study unit already met the BPT time-to-theatre criteria prior to introduction of the HFU, the significant reduction in time-to-theatre after implementation of the HFU demonstrates the value of ring-fenced theatre capacity, which would otherwise be faced with clinical pressures at a MTC to provide timely surgical care to fragility fractures. Failure to achieve BPT criteria has financial implications for hospital trusts, and the improvement in time-to-theatre demonstrated by our study has the potential to facilitate trusts achieve their BPT targets.^14^

The inclusion of distal femur fragility fractures within the FHF BPT will increase pressures on trusts to provide timely surgical treatment of these patients.^15^ Given the projected increase in the incidence of distal femur fragility fractures, the increased acute demand on theatre space may only be attainable with dedicated facilities such as HFUs with ring-fenced facilities for this cohort of medically frail and comorbid population.^16^

### Mortality rate

The significant reduction in 30-day mortality of 26.6% from 6.4% to 4.7% observed in this study is comparable with the results reported by Walton *et al* assessing the short-term results of HFU implementation. The overall 5-year mortality rate in the post-HFU cohort is lower than the national average and that reported in recent literature.^17^

The cause of the reduction in mortality is likely to be multifaceted and attributing the entire reduction to the HFU is not cogent. Notwithstanding, the reduction in time-to-theatre, co-location of the MDT and timely orthogeriatric physician support are all likely to contributory causal factors.^8,18–24^ Reductions in LoS in FHF patients have previously been shown to be down to the formation of an HFU.^25^

### Limitations and strengths

This study is limited predominantly by its retrospective nature, relying on existing databases. However, the databases used – the NHFD and local health records – are accepted as high-quality data sets because they are often required for the financial renumeration of trusts. This allows the authors to have confidence in the conclusions drawn.

The study’s time frame over 10 years aimed to capture sustained changes in outcome after formation of the HFU; however, we could have allowed confounding variables such as changes in hip fracture surgery anaesthesia to impact the outcomes being assessed.

With a population of ∼5,000 patients, this study represents one of the largest series assessing the outcomes of FHF in the UK. It also provides one of the longest consecutive series that illustrates the impact of service reconfigurations on patient care.

## Conclusion

This study demonstrates that a dedicated HFU, with ring-fenced theatres and a co-located MDT within a MTC, improves patient flow by reducing the time-to-theatre and LoS, while concurrently improving patient mortality at both 30 and 365 days post injury in a MTC setting. Therefore, the introduction of HFUs in these institutions may help to improve the flow of fragility fracture patients through the patient pathway.
